# Microbial Biosensor for Characterization of a Microorganism: A Review focusing on the Biochemical Activity of Microbial Cells

**DOI:** 10.3390/mi14040733

**Published:** 2023-03-25

**Authors:** Elena Emelyanova

**Affiliations:** Laboratory of Biosensors, G.K. Skryabin Institute of Biochemistry and Physiology of Microorganisms, Pushchino Center for Biological Research of the Russian Academy of Sciences, Prosp. Nauki 5, Pushchino 142290, Russia; elenvem@ibpm.pushchino.ru

**Keywords:** reactor microbial sensor, membrane microbial sensor, Clark-type oxygen electrode, oxygen consumption by microbial cells, constitutive and inducible enzymes, allosteric enzymes, substrate transport into microbial cells

## Abstract

Express assessment of the biochemical activity of microorganisms is important in both applied and fundamental research. A laboratory model of a microbial electrochemical sensor formed on the basis of the culture of interest is a device that provides rapidly information about the culture and is cost effective, simple to fabricate and easy to use. This paper describes the application of laboratory models of microbial sensors in which the Clark-type oxygen electrode was used as a transducer. The formation of the models of the reactor microbial sensor (RMS) and the membrane microbial sensor (MMS) and the formation of the response of biosensors are compared. RMS and MMS are based on intact or immobilized microbial cells, respectively. For MMS, the response of biosensor is caused both by the process of transport of substrate into microbial cells and by the process of the initial metabolism of substrate; and only initial substrate metabolism triggers the RMS response. The details of the application of biosensors for the study of allosteric enzymes and inhibition by substrate are discussed. For inducible enzymes, special attention is paid to the induction of microbial cells. This article addresses current problems related to implementation of the biosensor approach and discusses the ways how to overcome these problems.

## 1. Introduction

Sensors are analytical devices that help rapidly provide information about the studied analyte. In biosensors, a recognition element of a recognition part of the sensor is a biological component [1,2,3,4]. Microbial cells are used as the recognition element in microbial sensors [5]. If the measured response of substrate-recognizing microbial cells is a response typical of living cells (for instance, measurement of oxygen consumption by microbial cells in response to an analyte for a respiratory-based sensor), then only viable microbial cells can be used in these microbial sensors [6,7,8].

The important characteristics of a electrochemical respiratory-based microbial sensor is selectivity [2]. Selectivity is the ability of a biosensor system with high sensitivity to generate a response only for one of the components in a substance mixture (analyte). Selectivity of a microbial sensor depends on substrate specificity of the cells of a microbial culture used as a recognizing component of a biosensor (Hereinafter, a culture, which is the recognizing element of a biosensor, is called a culture-receptor by analogy to the nerve receptor, since the microbial cells sense the presence of the analyzed substance, analyte, and generate a corresponding response, which is further transformed by the biosensor transducer.) Substrate specificity of a microbial culture refers to biochemical characteristics of the culture. As to cells of culture which is used as a recognizing component of a microbial biosensor, substrate specificity is defined as the ability of cells to generate a signal in response to a substrate(s) specific for the culture-receptor. Even rather “simple” microbial cells have a broad spectrum of enzyme systems that are necessary for a wide range of metabolic reactions. Hence, on the one hand, the “disadvantage” of most microorganisms, the cells of which could be used as a recognizing element of a respiratory-based biosensor, is rather broad substrate specificity. As a result of this disadvantage, it is impossible to receive a culture response to only one component of the analyzed mixture. In this case, a rather laborious search among existing microorganisms, the creation of genetically modified cultures, or the optimization of detection conditions are required so that the biosensor detects only one substance in a mixture of substances. On the other hand, substrate specificity of one microorganism is insufficiently broad to determine the total content of various organic substances in a mixture that should be done while measuring biological oxygen demand (BOD) of wastewater. The use of a consortium of cultures is required for this purpose [9]. Thus, to ensure the selectivity of the determination of one substance in the analyte, the microbial sensor requires a number of improvements. However, the response to a certain substrate itself can characterize the metabolic features of a culture-receptor or the features of enzyme systems of this culture. Taken together, a laboratory model of a microbial sensor is a most suitable tool for the assessment of the biochemical activity of a microorganism (a culture-receptor), used as the recognizing biological component of a biosensor. Rapid assessment of the biochemical activity of microorganisms and activity of enzyme cell systems are very important not only for fundamental research but also for environmental monitoring (change in the activity of microorganisms-destructors in the presence of hazardous substances) and for the study of microorganisms.

For biochemical investigations, two types of microbial sensors can be applied: a reactor microbial sensor and a membrane microbial sensor [10]. These types of biosensors differ in the way the microbial cells communicate with the transducer. In the reactor microbial sensor (RMS), a suspension of intact microbial cells is used as a recognizing component; a receptor-element (microbial cells immobilized on supporter) is used in the membrane microbial sensor (MMS). Using the Clark-type oxygen electrode as a transducer (electrochemical determination), laboratory models of microbial sensors of both types were employed in our study to characterize features of metabolism in microorganisms. The Clark-type oxygen electrode made it possible to detect changes in oxygen concentration when a culture-receptor consumed oxygen. Therefore, specific features of metabolism and enzyme systems of microbial cells, which were oxygen-dependent (for example, a change in the activity of enzyme systems of microbial cells that was accompanied by a change in the intensity of culture-receptor respiration) were investigated. The purpose of the present work was to summarize the results of 20 years of research using laboratory models of respiratory-based electrochemical microbial sensors.

## 2. Principles of Formation and Operation of Biosensor Models

### 2.1. Formation of a Laboratory Model of RMS and the Principle of Determining the Cells’ Response

**Figure 1:**
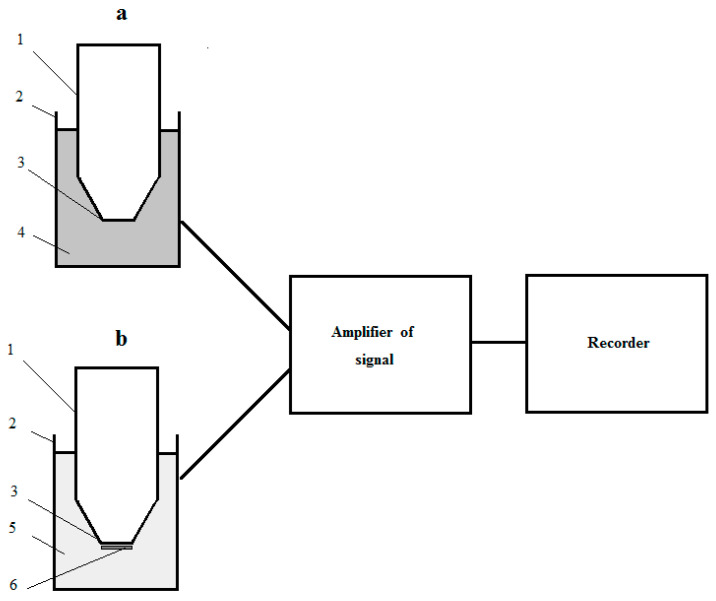
The design of laboratory models of the respiratory-based microbial sensor: (**a**) The recognizing part of RMS; (**b**) The recognizing part of MMS; 1—the Clark-type electrode; 2—measuring cell; 3—measuring surface of electrode; 4—suspension of culture-receptor cells in buffer solution; 5—buffer solution; 6—receptor-element (immobilized cells of culture-receptor).

The recognizing part of a laboratory model of a reactor microbial sensor consisted of a measuring cell with suspension of culture-receptor cells in buffer solution (a measuring solution) and the Clark-type oxygen electrode (a transducer) placed in the measuring solution. The measuring cell was equipped with a magnetic stirrer (the measuring cell was analogue of the ideal mixing apparatus). The magnetic stirrer ensured not only mixing, but also saturation of the measuring solution with air. The design of the recognizing part of the respiratory-based RMS and the full construction of the laboratory model of the biosensor is pictured in Figure 1. The transducer was connected to the signal amplifier (Ingold 531-04 O_2_ Amplifier, Instrumentation Laboratory, Milan, Italy); and amplified electrical signal of the transducer was registered with two coordinate recorders (XY Recorder-4103, Praha, Czech Republic).

**Figure 2:**
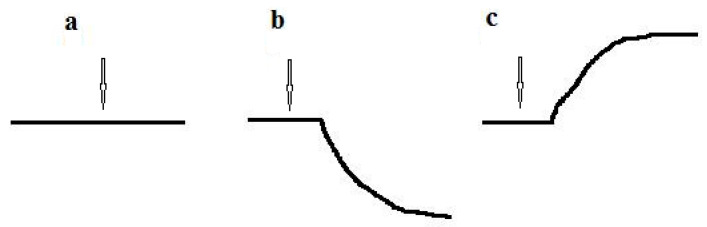
Analytical signal of the biosensor: (**a**) Basic endogenous respiration of culture-receptor cells in the absence of a substrate; (**b**) Response of culture-receptor cells to substrate, when cells’ respiration is activated after substrate injection; (**c**) Response of culture-receptor cells to substrate, when cells’ respiration is inhibited after substrate injection. The arrow shows the moment of substrate injection.

The response to the substrate for culture-receptor cells was measured at 20–22 °C and buffer solution was continuously stirred. Freshly harvested cells of the culture-receptor were suspended in an air-saturated buffer solution (wet weight of cells was 50 mg/mL) to obtain a suspension of intact cells of the culture-receptor. While working with microbial sensors, optimal results were obtained when microbial cells were suspended in a 50 mM K-Na phosphate buffer [11,12], which has been used since the past century [13,14]. Before measurements, a required amount of intact cell suspension was added to the buffer solution (the ratio of cell suspension volume to buffer solution volume was usually 1:4). Then, the endogenous (basic) respiration of the culture-receptor cells was stabilized in the absence of an exogenous substrate by mixing the suspension of microbial cells in the measuring cell. In this case, the recorder registered the basic respiration of cells due to the endogenous substrate; the analytical signal of the biosensor was a straight horizontal line (Figure 2a). To determine the culture-receptor response to the substrate, a substance was injected into the measuring solution. Approximately 5–10 seconds later, the culture-receptor cells generated a response to the injected substrate: the respiration of microbial cells changed in response to the injection of the substrate, leading to a change in the oxygen concentration in the measuring solution. The mixing was sufficient to ensure complete (ideal) mixing. Therefore, the oxygen concentration was the same all over the measuring solution. The oxygen concentration in the solution was measured using the Clark-type oxygen electrode (the transducer), which transduced a chemical signal (oxygen concentration) into an electrical signal (electrode current). The change in respiration of culture-receptor cells in response to the substrate (change in oxygen concentration) was proportional to the change in the electrical signal of the electrode (change in electrode current). Changes in the electrode current were registered using a recorder. The typical plot of a dependency of the biosensor signal change on time (the analytical signal of the biosensor) was hyperbolic [1] (Figure 2b,c). Usually, the respiration of microbial cells was activated in response to the presence of a substrate (for example, the curve obtained during signal recording was oriented as shown in Figure 2b). However, for some substrates, substrate injection resulted in inhibition of cell respiration. In this case, the recorded curve was oriented in the opposite direction (Figure 2c).

**Figure 3:**
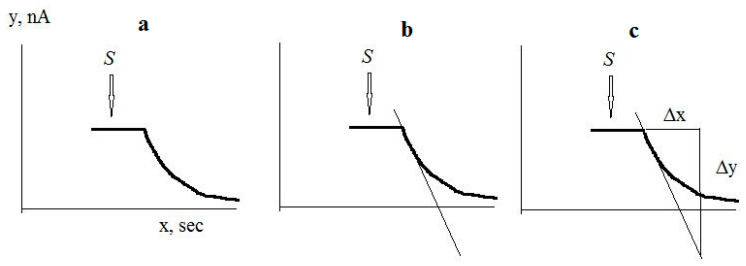
An example of calculation of the response to substrate: (**a**) Registration of the response to a substrate; (**b**) Drawing the tangent with the largest slope to the recorded response curve; (**c**) Construction of a right triangle, where the tangent is the hypotenuse. The arrow shows the moment of substrate injection.

The culture-receptor response to substrate was proportional to the initial maximum rate of electrode current change in response to substrate injection. The maximum rate of electrode current change was calculated as the first derivative of the electrode current change in response to substrate injection. An example of calculation of the response to substrate is shown in Figure 3: After registering the response to the substrate, a tangent is drawn with the largest slope to the recorded response curve. A right triangle is then constructed using the tangent as the hypotenuse, and the magnitude of the response is calculated as ∆y/∆x = dI/dτ in pA/s.

After registration of the response, the measuring cell was washed, then the measuring cell was filled with suspension of culture-receptor cells in buffer, and the next measurement of response was performed.

### 2.2. Formation of a Laboratory Model of MMS and a Principle of Cells’ Response Determination

To form a recognizing element (a receptor-element) of the recognizing part for a membrane microbial sensor, suspension of culture-receptor cells in a 50 mM K-Na phosphate buffer (wet weight of cells was 100 mg/mL) was prepared. To even out the intensity of metabolism in microbial cells, suspension was stored at + 4 °C for 12 h. Then the 10 μL of prepared suspension of microbial cells was immobilized on paper (Whatman glass paper was most often used [15]) by the method of physical sorption: the suspension was spotted (3 mm in diameter) onto a piece of paper (4 × 4 mm^2^). The formed receptor-element was air-dried for 30 min and was then used for formation of the MMS.

The receptor-element was fixed with nylon net on the measuring surface of the Clark-type oxygen electrode. The oxygen electrode with the attached receptor-element was a microbial electrode. The microbial electrode was placed in the measuring cell filled with the buffer solution. All this was a recognition part of the respiratory-based MMS, the design of which was shown in (a) of the Figure 1. As to the rest, the design of the laboratory model of the MMS was the same as for the RMS.

Measurement conditions and calculation of a culture-receptor response to substrate were the same as for the RMS. The difference was that the measuring cell was filled with a buffer solution without microbial cells. Cells of the culture-receptor were located only on the supporter of the receptor-element. A small receptor-element (4 × 4 mm^2^) was attached to the measuring surface of the oxygen electrode. Therefore, a change in the intensity of respiration of microbial cells in response to the injection of the substrate led to a change in the oxygen concentration in the vicinity of the measuring surface of the oxygen electrode. In addition, after measurement, the receptor-element was not replaced with a fresh one. The measuring cell with the microbial electrode was washed only to remove the injected substrate, and then the measuring cell was filled with a fresh buffer solution, and the next measurement was performed. When the receptor-element became unusable, it could be quickly replaced with a freshly prepared receptor element or with a receptor element that had been prepared in advance and stored at +4°C. Taking into account the time required for washing and stabilization of the basic (endogenous) respiration of the culture-receptor, on average, one measurement took about 15–20 minutes with minimal consumption of microbial biomass.

## 3. Applications of the Laboratory Model of Biosensor

### 3.1. Application of the RMS Model for Assessment of Biochemical Activity of Microbial Cells and Activity of Enzyme Systems of Microbial Cells

The laboratory model of the RMS was used for assessment of biochemical activity of *Rhodococcus opacus* 1CP actinobacterium (DSM 46757 and VKM Ac-2638). Inducible enzymes benzoate 1,2-dioxygenase (BDO), 3-chlorobenzoate 1,2-dioxygenase (3-CBDO) and phenol hydroxylase (PH) initiated metabolism of benzoate (BA), 3-chlorobenzoate (3-CBA) and phenol (ph), respectively, in cells of this culture. For cells grown in LB medium (BA-, 3-CBA- and ph-free medium), no responses to the substrates (BA, 3-CBA and ph) were recorded with the use of the RMS formed on the basis of uninduced cells (the absence of inducible enzyme initiating substrate metabolism in cells of the culture-receptor) [16,17,18]. Using MMS based on uninduced cells, a response to the substrate of inducible enzyme was registered. This response was caused by the process of substrate transport into microbial cells (see Section 3.2). Transport of substrate into uninduced cells of the culture-receptor occurred, but substrate metabolism was not initiated, as there were no inducible enzymes necessary for substrate metabolism. Consequently, the response recorded with RMS could not be caused by substrate transport into cells of the culture-receptor. However, responses to BA, 3-CBA, or ph were registered with the RMS formed on the basis of induced *Rhodococcus opacus* 1CP containing an enzyme that initiates substrate metabolism. Therefore, it was concluded that, for the RMS, the intensity of a response was stipulated only by the intensity of initial metabolism of substrate. Thus, the RMS can be employed to study the initial metabolism of substrate in culture-receptor cells and the activity of the enzyme initiating the metabolism of substrate. If the RMS is formed on the basis of uninduced cells of a culture (cells were grown on inducer-free medium), then the presence of a response to the substrate indicates metabolism initiated by a constitutive enzyme. For example, no response to ph was detected using the RMS based on uninduced cells of *Rhodococcus opacus* 1CP [16], which indicates the inducibility of the enzyme that initiates substrate metabolism in 1CP cells.

There is an analogy between the measuring cell of the RMS (suspension of culture-receptor cells is continuously stirred) and the ideal mixing apparatus for continuous cultivation of microorganism. For process of continuous cultivation, Dudina proposed the method of pulse addition for rapid identification of a growth-limiting substrate: rapid changes in pH and pO_2_ were observed in response to injection of a limiting substrate [19]. The method of pulse addition was applied by Emelyanova during continuous cultivation of yeast *Candida ethanolica* [20]. The observed response was similar to a response to substrate for culture-receptor suspension in the RMS. Due to the development of microreactor technology, a new type of the RMS can be developed on the basis of a microreactor, but the proposed pattern of flows needs to be improved [19].

### 3.2. Application of the Laboratory Model of the MMS for Assessment of Biochemical Activity of Microbial Cells

It is known that the response to substrate for immobilized cells of a culture-receptor of the MMS is caused by two processes. They are: a process of substrate transport into cells of a culture-receptor and a process of initial metabolism of substrate under the action of cell enzyme(s) [1]. Both processes contributed to the formation of the cells’ response to a substrate in the next two cases. The first case is when constitutive enzyme systems initiated processes, transport and metabolism. If cells of a culture-receptor were grown on a rich medium (substrate inducer-free medium ) and the response to a substrate was recorded using both the MMS and the RMS, then constitutive enzyme systems caused cells’ response to substrate [11,21]. The second case is when inducible enzyme systems caused cells’ response to substrate, i.e. when microbial cells were grown on a medium containing a substrate-inducer or were induced under non-growth conditions [22]. For MMS, when the intensity of the cells’ response to substrate was stipulated by the intensities of transport and metabolism, the response parameters (the maximum rate of cells’ response to substrate, *V_max_*, and the half-saturation constant, *S_0.5_*) were complex constants. These parameters characterized both processes. It is not yet clear how these complex parameters are formed.

If an inducible enzyme initiated substrate metabolism, then the response of the MMS formed on the basis of uninduced cells (inducible enzymes-free cells grown on an inducer-free medium) characterized the intensity of constitutive system(s) of substrate transport into cells of culture-receptor [16,22]. The half-saturation constant, obtained in the course of the mentioned MMS study, characterized the affinity of constitutive transport system(s) for the substrate. In this case, the MMS could be used to investigate the process of constitutive transport of substrate into microbial cells.

As mentioned in Section 2.1, the analytical signal of the biosensor is hyperbolic. The respiration of microbial cells was activated in response to the injection of the substrate, which led to a decrease in the oxygen concentration in the vicinity of the receptor-element. However, the substrate could inhibit culture respiration. If the sweep of the recorded signal occurred from top to bottom upon activation of respiration (Figure 2b), then during inhibition of respiration, the sweep went from bottom to top (Figure 2c) and *vice versa*. Thus, by orientation of the curve of response, it was possible to learn about the effect of the substrate on the respiration of the culture. So, in response to the injection of sodium dodecyl sulfate (SDS), respiration of *Pseudomonas rathonis* T cells was activated in comparison with endogenous respiration, in contrast to the response to the cardboard recycling wastewater [23].

In addition, the MMS was used to evaluate the “co-oxidation” of substrates. So, for the *Rhodococcus erythropolis* HL PM-1 actinobacterium, no culture response was registered in the presence of low concentrations of 2,4-dinitrophenol (2,4-DNP): 1 × 10^−12^–1 × 10^−5^ M. However, a fairly good response to ethanol (1 × 10^−3^ M) was observed for these actinobacterial cells. Moreover, the response to ethanol increased by 20–100% (depending on the concentration of 2,4-DNP) in the presence of low concentrations of 2,4-DNP [24]. The effect has been termed “co-oxidation” by analogy with the effect of cometabolism, when the metabolism of a difficulty utilized substrate was accelerated in the presence of a readily utilized substrate. In the case of true cometabolism, both substrates were metabolized in cells. In the case of “co-oxidation”, only ethanol was true respiratory substrate, but 2,4-DNP stimulated this respiration. By stimulating the culture response to ethanol in the presence of 1 × 10^−12^–1 × 10^−5^ M of 2,4-DNP, concentration of 2,4-DNP could be determined [25].

### 3.3. Application of the Laboratory Models of the Microbial Sensors for Study of Inhibition by a Substrate

For the MMS and, for example, fusaric acid (FA), the classical curve for a toxic substrate was obtained when a decrease in respiration of microorganism (inhibition by high concentrations of substrate) was observed. The inhibition-threshold substrate, FA, concentrations were determined for *Fusarium oxysporum* f. sp. *vasinfectum* Coll and *Bacillus subtilis* 128. Their magnitudes were close to the magnitudes determined by other researchers using a classic microbiological method. For *Fusarium oxysporum* f. sp. *vasinfectum* Coll, the constant of inhibition by substrate (*S^si^_0.5_*) was also determined [26].

Using the RMS formed on the basis of induced cells of actinobacterium *Rhodococcus opacus* 1CP, constants of enzyme inhibition by substrate were calculated for allosteric [27] enzymes (BDO and 3-CBDO) of the actinobacterium. These enzymes catalyze the reactions, in which oxygen is involved. BDO and 3-CBDO are enzymes of the complex structure; they could not be isolated in pure and stable form without loss of the enzymes’ activity. That’s why the activity of these enzymes is measured indirectly in intact enzyme-containing microbial cells (induced cells) as the change in oxygen consumption by cells in response to the enzyme substrate [28]. One of the modifications of BDO or 3-CBDO activity measurement is the polarographic determination with intact cells; the polarographic determination is similar to the measurement with the RMS formed on the basis of intact cells containing BDO or 3-CBDO. Not only the activities of BDO or 3-CBDO enzymes, but also the constants of enzyme inhibition by substrate-inhibitor, were determined with the RMS formed on the basis of *Rhodococcus opacus* 1CP induced cells [17,18,29]. For the types of inhibition described by Dixon et al. [30], the calculation of constants of enzyme inhibition by substrate stems from the knowledge of the mechanisms of inhibition. However, there exist significantly more types of inhibition than Dixon et al. reported [30], the mechanisms of not all processes are established. In this case, the method of vector representation of enzymatic reactions (VPER method) proposed by Krupyanko is required [31]. According to Krupyanko method, the type of enzyme inhibition by substrate-inhibitor was identified with comparison of the relative position of the graphs of 1/*V* vs. 1/*S* dependencies in the presence and absence of a substrate-inhibitor (where *V* is the rate of enzymatic reaction, determined in our case with the RMS, and *S* is the initial concentration of substrate of an enzyme) and with determination of coordinates of the point of intersection for 1/*V* vs. 1/*S* graphs [17,18]. For determination of a constant of BDO inhibition by 2-chlorobenzoate, the MMS formed on the basis of *Rhodococcus opacus* 1CP induced cells was also applied.

**Figure 4:**
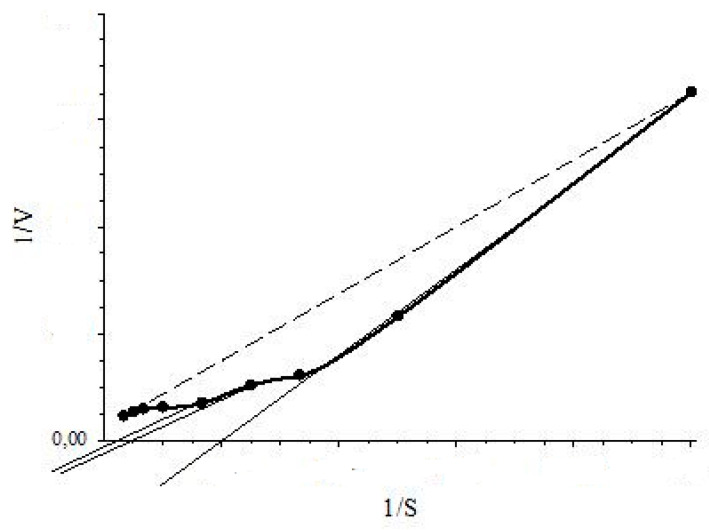
Intersection with the 1/*S* axis for the tangent to the concave curve (positive kinetic cooperativity by substrate, the Hill kinetics): dotted line is 1/V-1/S plot for the Michaelis-Menten dependency, the solid thick curve is the 1/V-1/S plot for the Hill dependency (positive cooperativity).

As mentioned above, inhibition of BDO, 3-CBDO and PH by substrate-inhibitors was studied with the RMS. It should be noted that the kinetics of these enzymes deviated from the classic hyperbolic saturation kinetics (a typical Michaelis-Menten kinetics). The sigmoidal dependency of *V* on *S* (Hill kinetics) and positive kinetic cooperativity by substrate were observed for BDO [17] and 3-CBDO [18]. Linearity of a 1/*V* vs. 1/*S* dependence confirms that a *V* vs. *S* dependence was hyperbolic and *vice versa*. For an allosteric enzyme, concavity of a 1/*V* on 1/*S* curve implied positive kinetic cooperativity by substrate and convexity indicated negative kinetic cooperativity by substrate for Hill kinetics [32]. Therefore, linearity or concavity/convexity of the plot of 1/V vs. 1/S indicates Michaelis-Menten or Hill kinetics, respectively. Moreover, while determining the type of inhibition, plotted graphs of 1/*V* vs. 1/*S* dependencies could point to the type of kinetics for an enzyme. As shown in Figure 4, drawing a tangent to the concave curve (plot of 1/V vs. 1/S) in the range of 30–100% of 1/S (substrate inhibition study interval), a straight line will intersect the 1/S axis at positive values. This position of the point of intersection will indicate the Hill kinetics: positive kinetic cooperativity by substrate. The Hill kinetics was showed for constitutive systems of ph transport into immobilized of *Rhodococcus opacus* 1CP cells (with the MMS) [16] and in study of 3-CBDO inhibition by 4-CBA (with the RMS) [18]. For the classic Michaelis-Menten kinetics, a graph of 1/*V* vs. 1/*S* dependency is a line that intersects the 1/*S* axis in the area of negative values.

## 4. Preparation of the Culture-Receptor

### 4.1. Growth Conditions for the Microorganism (A Culture-Receptor)

To form the recognizing part of the biosensor, biomass of culture-receptor grown on agar or liquid nutrient medium was used. When the response of microbial cells to the substrate depended on the activity of constitutive enzyme systems, the culture-receptor was grown on a rich medium, for example, on agarized Luria-Bertani (LB) medium and malt agar [11,26], depending on the culture used, or in appropriate liquid media. When the activity of inducible enzyme systems was evaluated, or the intensity of a response to the substrate for the culture-receptor depended on the activity of the inducible enzyme, then the culture was grown on a mineral medium containing the substrate of enzyme as a carbon source. The substrate of enzyme was a substrate-inducer. This substrate induced the synthesis of the inducible enzyme in the cells of the culture-receptor: the content of the enzyme in the cells of the microorganism became higher than the minimum baseline characteristic of uninduced cells. For example, benzoate, 3-chlorobenzoate, or phenol were substrate-inducers for enzymes benzoate 1,2-dioxygenase, 3-chlorobenzoate 1,2-dioxygenase [22] or phenol hydroxylase [16], respectively.

If necessary, the induction of enzyme systems was also carried out under non-growth conditions.

### 4.2. Induction of Enzyme Systems of Culture-Receptor Cells under Non-Growth Conditions

Induction by a substrate-inducer is necessary in the study of processes catalyzed by inducible enzyme systems. The induction of enzyme systems in the cells of the culture-receptor could be carried out not only when growing a microorganism in the presence of a substrate-inducer, but also under non-growth conditions.

For the induction of the culture-receptor in the case of the MMS, the receptor element was not removed from the electrode surface. To carry out induction, a substrate-inducer was injected into the buffer solution, where there was a microbial electrode. The receptor-element containing the immobilized cells of the culture-receptor was kept in this solution with stirring for at least a day. To complete the induction process, the measuring cell and microbial electrode were washed to remove the remaining substrate-inducer.

When working with the RMS, induction could be carried out in suspension of intact cells prepared for the formation of the RMS. The substrate-inducer was added to suspension, and suspension was kept with stirring for at least 24 h. Because the receptor culture cells were suspended in a buffer solution but not in a nutrient solution, the induction conditions were non-growth. After completion of induction, microbial cells were isolated from the solution by centrifugation and suspended in a buffer solution. The resulting suspension of intact induced cells was used for measurement.

Induction, even under non-growth conditions, led to a significant increase in activity measured with the RMS [33], even in cells after 4 months of storage in a buffer solution [34]. Under non-growth conditions, protein synthesis occurred in microbial cells in the presence of a substrate-inducer, which was revealed by study in the presence of a protein synthesis inhibitor (chloramphenicol) [17]. It is worthy of note that the concentration of a substrate-inducer influenced the state of the induced enzyme of culture. So, it was found that the concentration of the substrate, BA, in growth medium influenced the activity of BDO of *Rhodococcus opacus* 1CP. With increase of BA concentration, kinetics of the process catalyzed by BDO changes from the sigmoidal saturation Hill kinetics to the hyperbolic saturation Michaelis-Menten kinetics [35]. Using MMS, it was shown that induction led to classical Michaelis-Menten kinetics for the pair of Bacillus subtilis-fusaric acid [26].

## 5. Conclusions

In summary, laboratory models of the RMS and MMS were convenient tools for express evaluation of biochemical processes in cells of a culture-receptor. Table 1 summarizes the results of using RMS and MMS in biochemical studies.

Obviously, the study of any biochemical process in vitro is not 100% analogous to the same process occurring in vivo. There are differences between them, and sometimes very significant ones… However, it is impossible to exclude laboratory research methods. So, why not to use a quick and easy-to-use biosensor approach for express assessment of biochemical features of cultures and enzymes?

## Figures and Tables

**Table 1:** The use of laboratory models of respiratory electrochemical microbial sensors for biochemical research.

Type of Microbial Sensor	Processes Caused Biosensor Response to Substrate ^*^	Application of Biosensor Models for Investigation
RMS	Initial metabolism of substrate in cells of culture-receptor	- Activity of enzyme initiating metabolism of substrate - Substrate inhibition of enzyme initiating metabolism - Indication of constitutiveness (for uninduced cells) or inducibility (for induced cells) of enzyme initiating metabolism - Estimation of the type of kinetics for enzyme-substrate interaction
MMS	Transport of substrate into cells of culture-receptor and ** Initial metabolism of substrate in cells of culture-receptor	- Evaluation of the process of constitutive transport of substrate into uninduced cells *** - Assessment of complex constants characterizing both processes: transport and metabolism of substrate - Substrate inhibition of both processes: transport and metabolism of substrate - The influence of the substrate on the microorganism (culture-receptor)

* In the presence of enzyme systems, which caused the response of the biosensor (cells of culture-receptor) to the substrate. ^**^ In the presence of enzyme systems that cause both processes.^***^ In the absence of constitutive enzyme initiating metabolism of substrate in cells of culture-receptor.

## Data Availability

The data are contained within the article.
